# Application of transiliac approach to intervertebral endoscopic discectomy in L5/S1 intervertebral disc herniation

**DOI:** 10.1186/s40001-017-0254-0

**Published:** 2017-04-04

**Authors:** Jiayue Bai, Wei Zhang, Yapeng Wang, Jilong An, Jian Zhang, Yapeng Sun, Wenyuan Ding, Yong Shen

**Affiliations:** The Third Hospital of Hebei Medical University, 139#Ziqiang Road, Shijiazhuang, Hebei China

**Keywords:** Lumbar degenerative disease, Transiliac approach, Minimal invasive surgery, Percutaneous transforaminal endoscopic discectomy

## Abstract

**Objective:**

To discuss the significance and the short-term effect of bone puncture technique in transiliac approach to intervertebral endoscopic discectomy for the treatment of L5/S1 intervertebral disc herniation.

**Methods:**

Nineteen patients were diagnosed as L5/S1 disc herniation and treated using transiliac approach to endoscopic discectomy (group I), and 20 patients were diagnosed as non-L5/S1 disc herniation and underwent conventional approach (group R). Leg pain was evaluated by VAS. MacNab ratings of the last follow-up were recorded to evaluate early clinical efficacy, and postoperative complications were recorded to evaluate surgical safety. The imaging changes of the patients 3 months after surgery were observed.

**Results:**

One patient in group I, who felt abnormal in nerve roots, underwent symptomatic treatments, such as rehydration and hormone, and the abnormalities disappeared 3 days after treatment. There were no significant significances in operative time and intraoperative fluoroscopy times between groups I and R (*p* > 0.05), but there was a higher tendency in group I. The VAS scores of post-operation were significantly lower than that of pre-operation in the two groups (*p* < 0.05), but there were no significant differences between the two groups (*p* > 0.05). The MacNab score of the last follow-up showed excellent rate (95%) and good rate (90%) in groups I and R, respectively.

**Conclusions:**

Bone puncture-combined transiliac approach to intervertebral endoscopic surgery could locate iliac puncture point individually, and establish a good iliac channel, which is safe, effective, and minimally invasive.

## Background

Recently, percutaneous transforaminal endoscopic discectomy (PTED) is widely used in the treatment of lumbar disc herniation, which obtains good effects [1–3]. However, it is difficult for part of the patients using this surgery, who has high iliac crest, especially in the segments L5–S1, because of the block of iliac crest. Therefore, for spine surgeons, it is still a thorny problem for these patients using intervertebral endoscopic surgery.

Choi [4] and Rueteen [5] utilized endoscopic surgery to treat the patients with high iliac crest lumbar disc herniation, including high central, next to the central and prolapsed, through transforaminal approach which achieved good clinical result. But there was a higher incidence of nerve injury in the early period, and this approach was difficult to handle the lateral and the extreme lateral disc herniation [6]. Chio [7] reported two cases of transiliac approach to intervertebral endoscopic discectomy in 2009, which enriched the surgical approach to the spinal endoscopic discectomy. However, there were few reports about the surgical treatment of L5–S1 disc herniation with high iliac crest, and its safety and efficacy remained uncertain. In addition, the key of the success for this surgical approach was how to precisely locate the puncture point on the ilium and establish iliac channel in good position. To solve this problem, an innovative application was built through the bone puncture technology to establish iliac channel, then remove intervertebral disc through endoscope, and the early clinical results were satisfactory.

The present study was approved by the hospital ethics committee, and the clinical data of the treated 19 patients with L5/S1 disc herniation were analyzed. The clinical efficacy of bone puncture technique in transiliac approach to intervertebral endoscopic discectomy for the treatment of L5/S1 intervertebral disc herniation was investigated, and the surgical skills were summarized to provide reference for clinical practice.

## Materials and methods

### Subjects

From March 2014 to September 2015, 19 patients (10 males and 9 females) with L5/S1 disc herniation were treated using transiliac approach to intervertebral endoscopic discectomy (group I) with an average age of 38 years (aged 24–57 years) and a course of 7.8 months (6–23 months). Twenty patients (12 males and 8 females) with non-L5/S1 disc herniation, who were chosen as control group, underwent conventional transforaminal approach endoscopic discectomy (R group) with an average age of 41 years (aged 25–51 years) and a course of 13.8 months (8–19 months). All patients had various degrees of radiating pain in unilateral lower limb nerve root. This study was approved by the ethics committee of the Third Hospital of Hebei Medical University, and all patients signed informed consent.

### Diagnostic criteria

#### Inclusion criteria

Group I: (1) Single segment with L5/S1 disc herniation in high iliac crest (the definition of high iliac crest [8]: supine anteroposterior X-ray showed the iliac crest were located above L5 pedicle); (2) all patients had various degrees of radiating pain in unilateral lower limb nerve root; (3) patients took conservative treatment for at least 6 months and had poor effect and neurological impairment; (4) the X-ray examination of preoperative lumbar spine power showed no occurrence of lumbar instability; (5) scheduled for PTED surgery.

Group R: (1) Single segment without non-L5–S1 segment disc herniation in high iliac crest; (2) all patients had various degrees of radiating pain in unilateral lower limb nerve root; (3) patients took conservative treatment for at least 6 months and had poor effect and neurological impairment; (4) the X-ray examination of preoperative lumbar spine power showed no occurrence of lumbar instability; (5) scheduled for PTED surgery.

#### Exclusion criteria

(1) Patients had significant degenerative lumbar deformity, instability, tumor, infection, calcification of intervertebral disc, lumbar spinal canal and intervertebral foramen bony stenosis preoperatively; (2) patients had severe prolapse or dissociate of nucleus pulposus; (3) patients had bilateral neurological symptoms disc herniation or ipsilateral lumbar spinal stenosis, and intervertebral disc calcification seriously; (4) protruding sections was not affected by the iliac crest; (5) patients had other serious illness or allergy to metal, and others conditions that were not suitable for PTED (6) patients who had incomplete or lost data in follow-up period.

### Methods

#### Located the puncture point preoperatively

**Fig. 1:**
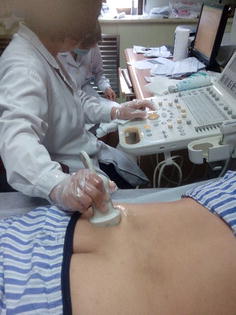
The vascular malformation of the superior gluteal artery were checked by routine preoperative hip vascular ultrasound

**Fig. 2:**
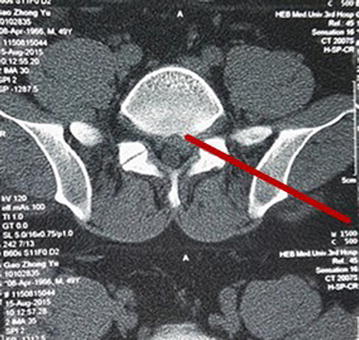
According to preoperative CT of the surgery segments to plan a good channel approach to foramen (the *red line*)

**Fig. 3:**
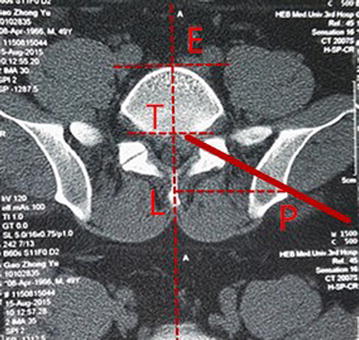
*P* is the ilium puncture points, *L* is the projection of coronary line above the median sagittal line. Measure the length ratio of ET and TL

The vascular malformation of the superior gluteal artery was checked by hip vascular ultrasound before surgery (Fig. 1). According to preoperative CT of the surgery segments, a good channel approach was planned (Fig. 2). The vertical line was drawn from the intersection of the auxiliary line of this approach and ilium to the center of the vertebral sagittal line, and finally intersected with the median sagittal line in spinous process (Fig. 3). The intersection was the intraoperative iliac puncture point in the lateral X-ray film. The distance of the intersection to the posterior vertebral body was measured and compared to the anteroposterior diameter of the vertebral bodies.

#### Establish an iliac channel by locating iliac puncture point individually

**Fig. 4:**
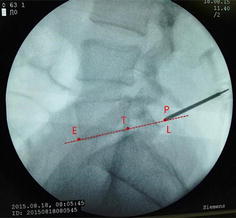
Adjust the needle puncture to make sure the two points coincide (*P* and *L*)

**Fig. 5:**
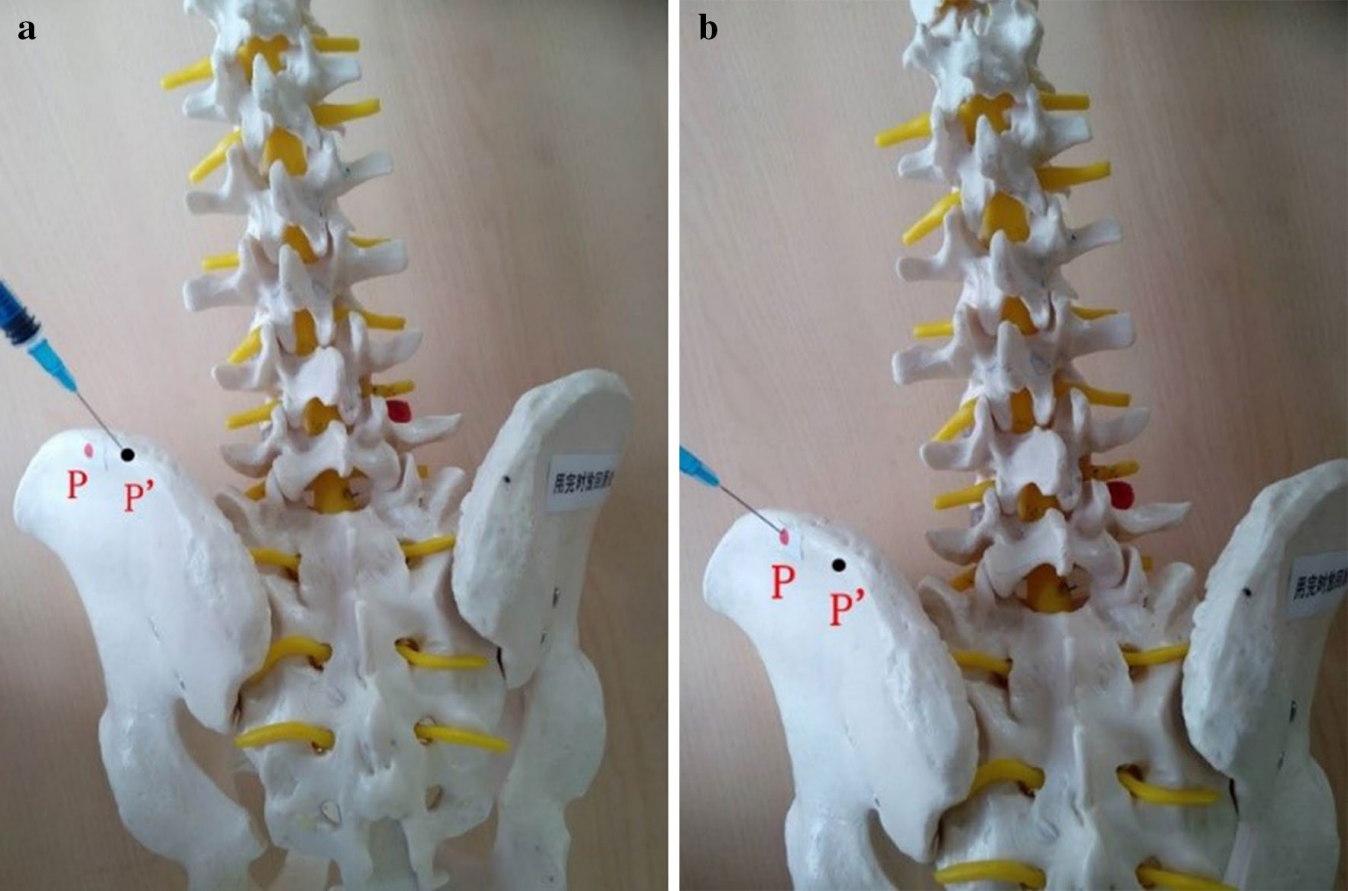
**a**, **b** The diagram of location of ilium puncture point: P’ is error puncture point, and P is the real puncture point (*left*, before the adjustment; *right*, after the adjustment)

**Fig. 6:**
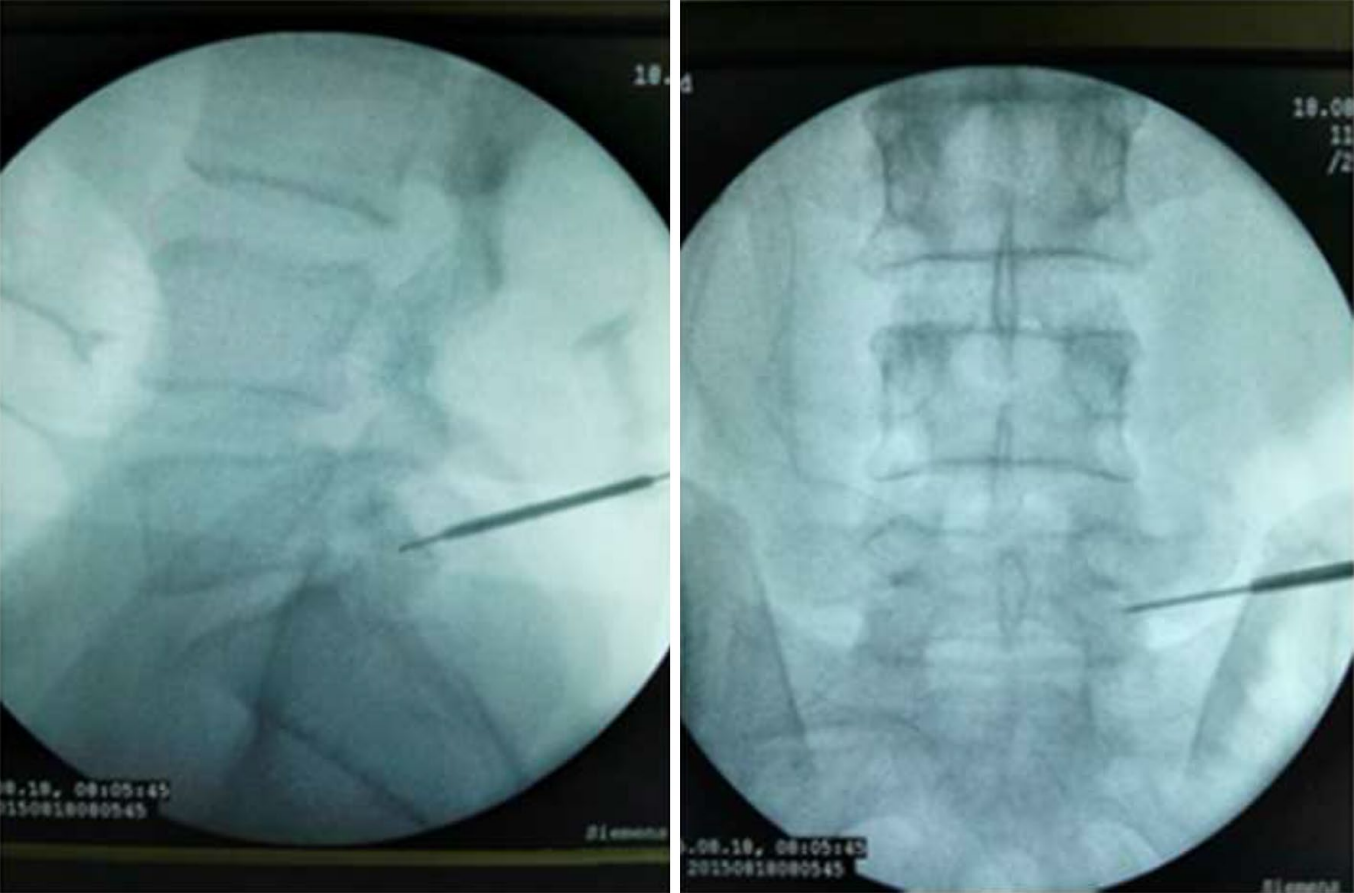
Needle into ilium, take out the needle core, and implant thread

**Fig. 7:**
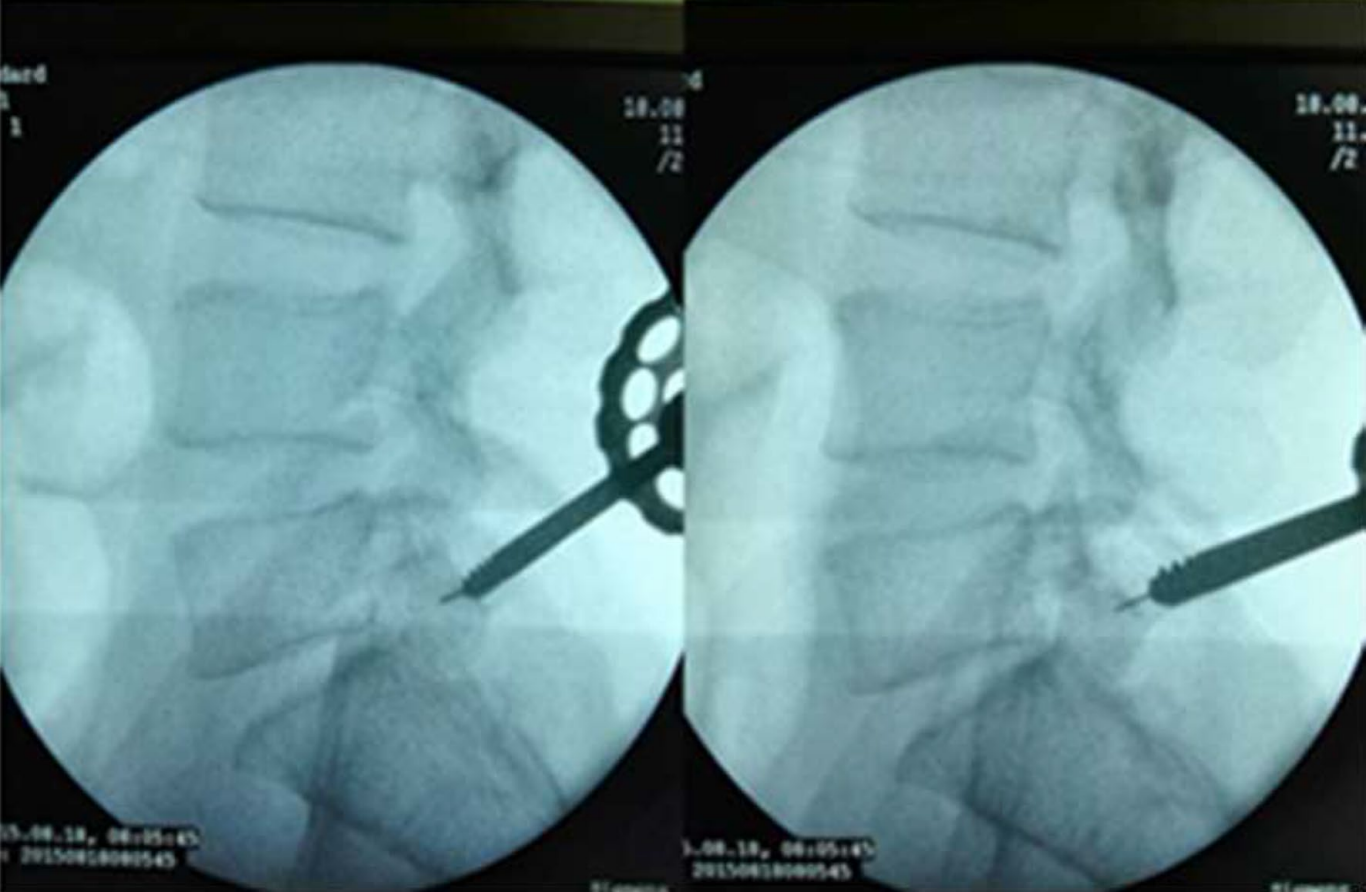
Using expanding drill enlarge iliac channel diameter to 7.5–10 mm

**Fig. 8:**
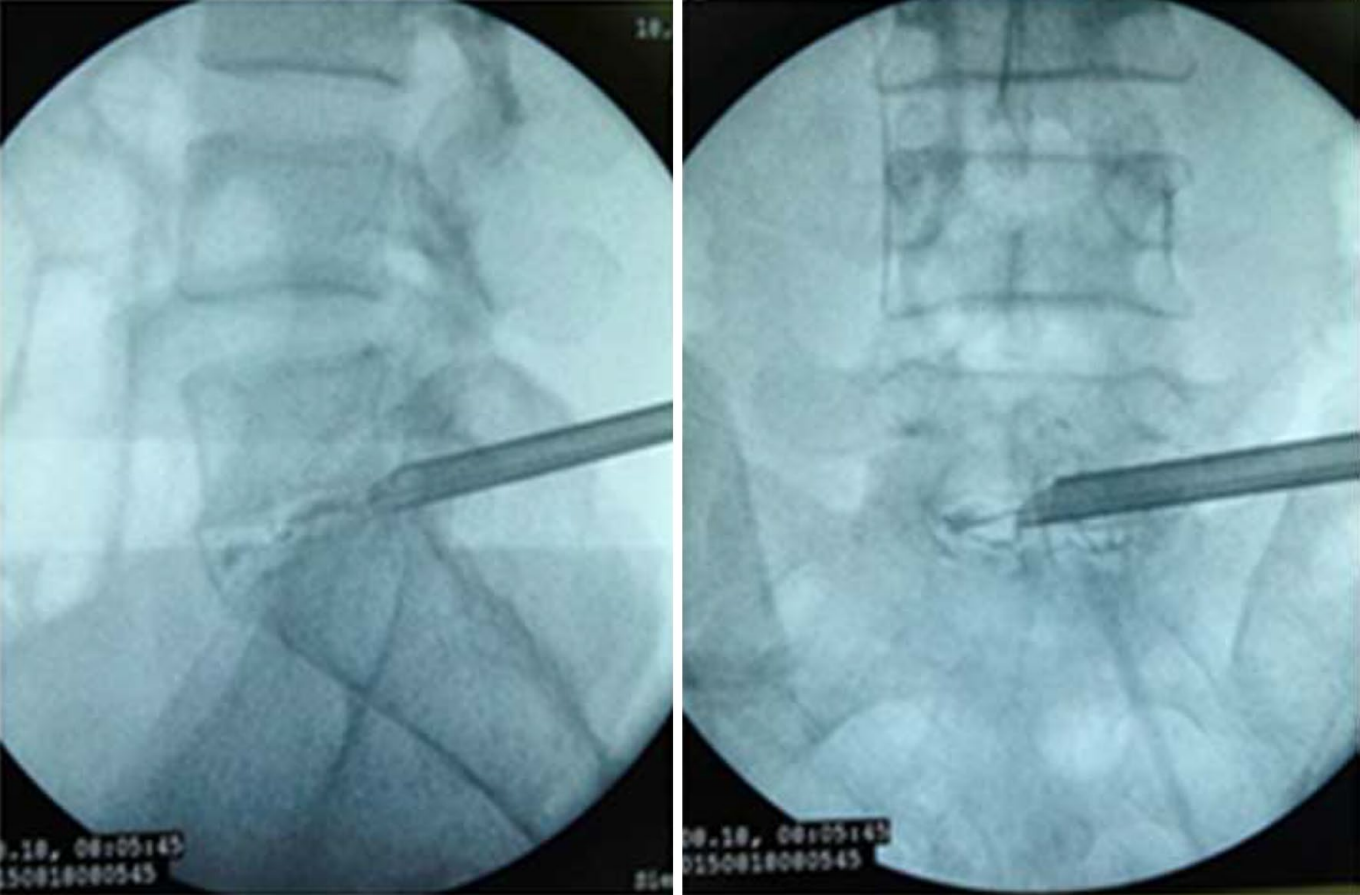
Through the ilium channel install a good working channel

The patients took lateral position or prone position, and skin puncture site is determined according to a conventional positioning method which was assisted by the C-arm fluoroscopy [7]. Skin puncture point was local anesthetized, and then bone puncture needle was used to puncture the skin parallel to the ilium. The puncture needle was adjusted to right above the iliac point to reach the preoperative puncture position (Figs. 4, 5). After the ilium was punctured, the pin core was removed and the guide wire was implanted along the needle (Fig. 6). Then, the needle was removed, and bone channel was expanded to the diameter of 7.5–10 mm by reamer implanted along the guide wire (Fig. 7). A conventional needle was inserted into the surgical site through the channels and intervertebral puncture, and then the guide wire was implanted along the needle. Dilatation catheter was implanted along the guide wire, and the soft tissue around the guide wire was expanded too. Finally, a working channel with good location and intervertebral foramen were implanted along the guide pipe (Fig. 8).

#### Operation under endoscopic

**Fig. 9:**
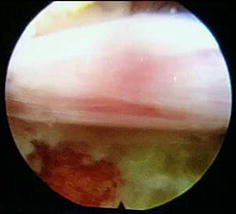
The surface of nerve root and full of blood vessels

Herniated nucleus pulposus were removed in the guidance of intervertebral foramen, while cleaning debris, osteophytes and hyperplasia tissue of vertebra posterior border which oppress nerve root, and using bipolar electrode radiofrequency ablation to stop bleeding. Dural sac pulsated independently when nerve root released completely, and the blood supply was significantly improved on the surface of the nerve root (Fig. 9). Then, the nerve root was reset. Intraoperative straight leg raising test showed that the nerve root could slip freely after being pulled. At the end of surgery, the perceived pain, soreness and numbness reduced, even disappeared. Make sure there were no bleeding and residual fragments in the field of vision by rotating working column, and then remove endoscopic and work column. Incision sutured one needle.

### Postoperative management

Patients wearing waist could get out of bed after reposed for 1–2 days, and do some exercises (waist and back muscle function and straight leg raising exercise). After 1 week, patients could do some light manual labor. The dehydration, bleeding and neurotrophic drugs were used according to the specific circumstances of the patient. Bony structure was studied by postoperative reviews of three-dimensional reconstruction of lumbar CT (computed tomography), lumbar MRI (magnetic resonance imaging) and pelvis CT.

### Effective evaluation criteria

A third party, who was not involved in this study, evaluated the leg pain by visual analog scale (VAS) before surgery, immediately after the surgery and 12 months after surgery. Early clinical efficacy of patients was evaluated by MacNab ratings in the last follow-up: (1) excellent, patients without low back pain and limitation of activity; (2) good, patients with waist or leg pain occasionally, but it did not affect their work and life; (3) average, lumbar function of patients had improved, but there was intermittent pain, and the patients had to change work and life; (4) poor, there was no improvement in pain and function. Postoperative complications were observed and recorded. We got access of follow-up for 19 patients for 12 months.

### Statistical method

The data were expressed as mean ± SD (standard deviation) for at least three independent experiments. SPSS 17.0 software was used for statistical analyses, and the Student’s *t* test was used to determine the significance of differences between two groups. *p* value <0.05 was considered statistically significant.

## Results

**Fig. 10:**
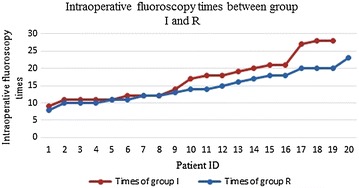
The line chart of intraoperative fluoroscopy times between groups I and R

All patients had a successful operation. There was no significant significance in operative time, blood loss, length of stay, VAS early postop and intraoperative fluoroscopy times between groups I and R (Tables 1, 2), but group I had a higher tendency (Fig. 10). The VAS scores of post-operation were significantly lower than pre-operation in the two groups, and there was no significant difference between groups (Table 1). According to the criteria of the MacNab score of the last follow-up, in group I, 14 cases were excellent, 4 cases were good, 1 case was average and no case was poor, and the excellent and good rate was 94.7% (18/19). In group R, 13 cases were excellent, 5 were good, 2 were average and no case was poor, and the excellent and good rate was 90% (18/20). One patient in group I, who felt abnormal in nerve root, underwent symptomatic treatments, such as rehydration and hormones, and the abnormalities disappeared 3 days after treatment. Postoperative CT scan and 3D (three-dimensional) reconstruction displayed that the diameter of iliac channel was consistent with the expanding drill, and there was no neurological damage associated with iliac bone puncture channel by clinical observation, including superior gluteal artery, the lowest lumbar spinal artery spinal branch vessels and femoral nerve, obturator nerve, lumbosacral trunk, as well as the iliac fractures, bone fractures and other injuries in addition to the ilium channel.

**Table 1:** The data of two groups M (P25, P75)

	Time of operation (min)	Intraoperative fluoroscopy times	Preoperative VAS	12 months postoperatively VAS	Comparison between before and after operation
Group I	86 (63, 92)	17 (11, 21)	8 (7, 9)	1 (0, 1)	*p* = 8.26 × 10^−8^
Group R	80 (55, 100)	14 (11, 18)	8 (7, 8)	1 (0, 1)	*p* = 3.51 × 10^−8^
Intergroup comparison	*p* = 0.899	*p* = 0.296	*p* = 0.641	*p* = 0.694

**Table 2:** The data of blood loss, length of stay and VAS early postop between two groups

	Blood loss (ml)	Length of stay (*d*)	Preoperative VAS	Immediate postoperative VAS
Group I	14 (11, 17)	5 (4, 5)	8 (7, 9)	2 (1.5, 2.5)
Group R	15 (8, 19)	4 (4, 5)	8 (7, 8)	1.5 (1, 2.5)
Intergroup comparison	*p* = 0.866	*p* = 0.517	*p* = 0.641	*p* = 0.583

## Discussion

Lumbar intervertebral disc herniation is a common disease of spine surgery with a high incidence rate. When conservative treatment fails, surgery is often needed [9]. Recently, endoscopic spine technology has developed due to the development of minimally invasive spine surgery. Percutaneous endoscopic lumbar discectomy (PELD) attracted widespread attention for less trauma, less bleeding, low cost, rapid recovery, etc. [10, 11]. Yeung [12] reported that there were 307 cases with lumbar disc herniation treated by YESS technology, and the patients were followed up for more than 1 year with a success rate of 89.3%. Hoogland [13] reported there was a 90% success rate for patients with lumbar disc herniation who were treated with TESSYS (transforaminal endoscopic spine system) technology and followed up for 2 years after surgery. The success rate of recurrent patient was about 85%, and below 3% in early period.

Scholars agreed that the key to successful intervertebral endoscopic surgery was to establish iliac channel in good position which could get to the lesion directly. However, the block of high iliac crest in some patients limited the angle of channel implantation. And the block of bone structure (lumbar bone hyperplasia and hypertrophia of transverse process) made it difficult to implant working channel in a good position in the lower lumbar disc herniation treatment (especially L5/S1 segment), thus resulting failure of puncture in many patients [1–3]. Therefore, Choi [4] and Rueteen [5] used the interlaminar approach endoscopic surgery to treat the iliac crest with high central, next to the central and prolapsed lumbar disc herniation, and achieved good clinical results in some patients. But in the early period, interlaminar approach carried a higher incidence of nerve injury, and it is difficult to eliminate lateral and far lateral protrudes. Then, some scholars [14] noted that the interlaminar approach was not suitable in the following conditions: (1) the presence of central spinal stenosis (<10 mm) confirmed by intervertebral disc CT and MRI; lateral recess stenosis (<3 mm) or small articular process hyperplasia; (2) small laminar space (horizontal or vertical spacing <7.5 mm); (3) intervertebral disc prolapse or dissociated to the head, or extreme lateral intervertebral disc herniation; (4) patients had surgery at the same segment twice.

Chio [14] reported that two cases with high iliac crest L5–S1 disc herniation were treated by transiliac approach to endoscopic discectomy, which provided a new idea for minimally invasive spine surgeons. But there were few reports about this approach (research only reported in the one case), so the safety and effectiveness needed to be further studied. Moreover, how to accurately locate the iliac puncture point and establish iliac channel in good position were keys to the application of the surgical approach.

Scholars agreed that the operation had potential for injuring the superior gluteal artery, cutaneous nerve and sacroiliac joint [15, 16]. Lu [15] found the average distance between the superior gluteal nerve and superior gluteal artery to iliac crest was 68.8 and 62.4 mm after studying the anatomy of cutaneous nerve in the posterior superior iliac, which indicated the possibility of establishing a 10-mm diameter channel from ilium to L5–S1 intervertebral disc. Osman [16] further demonstrated the feasibility of the transiliac approach to intervertebral endoscopic surgery after the directly drilling on the body. Chio had the clinical applications of lunging ilium on this basis.

**Fig. 11:**
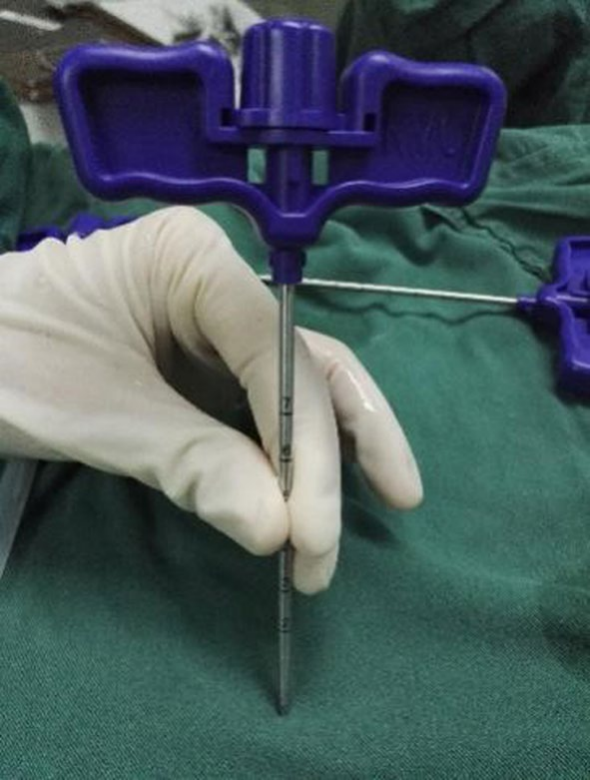
The adjustable needle for bone puncture

**Fig. 12:**
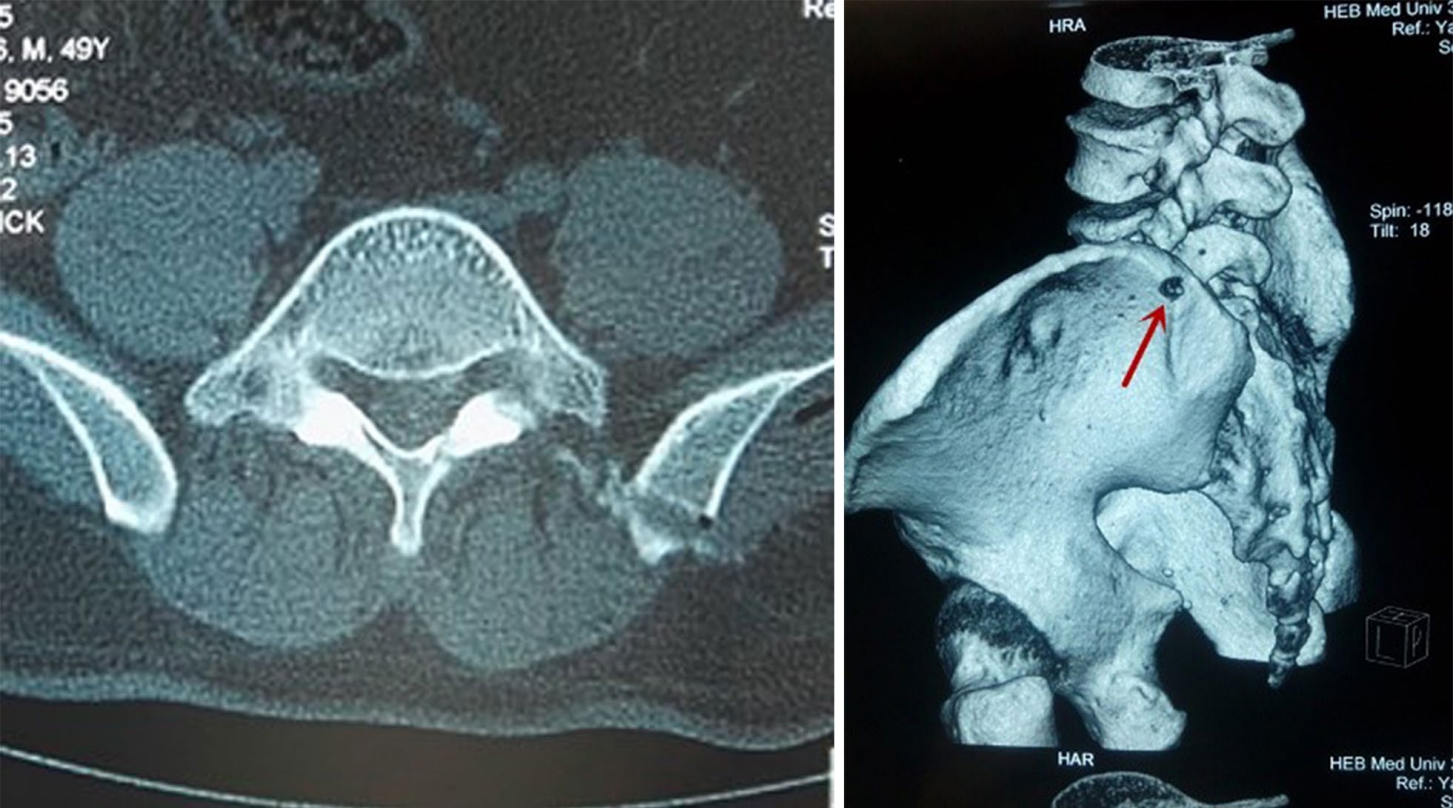
The CT ilium channel postoperatively. *Left* The vertebral CT scans after transiliac approach to intervertebral endoscopic discectomy and the bone channel was seen clearly. *Right* The vertebral CT three-dimensional reconstruction after transiliac approach to intervertebral endoscopic discectomy. Where the *arrow* is located is the bone channel

For precisely locating the iliac puncture point and establishing transiliac channels, we put forward the technique of bone biopsy which combined preoperatively individualized localization with bone puncture needle intraoperatively. An optimum individual channel was designed depending on protruding parts of the patient by preoperative CT (Fig. 2), and good iliac puncture site and lateral projection point in the median sagittal line were found on the basis (Figs. 4, 5). The distance of projection point to vertebra posterior border and the ratio of anterior and posterior vertebral body diameter were measured and calculated. The angle and position of the needle could be adjusted during the surgery assisted with lateral C-arm fluoroscopy (Figs. 4, 11). Gun found that the iliac sclerotin was thin, and the resistance of puncture was small [7], which would not cause massive hemorrhage, and this study also confirmed this opinion. Hence, the strength of puncture should not be too large, so as to avoid unnecessary trauma. The angle of the sagittal plane of the needle was also adjusted depending on the position of spinal nucleus pulposus prolapse to ensure the implanted working channel reached the position of moving nucleus. After the ilium was successfully punctured, the bone drill was used to expand the diameter of iliac channel to 7.5–10 mm (Fig. 7), and dilating catheter was used to expand surrounding soft tissue and implanted working channel (Fig. 8). In this step, the diameter of iliac channel was larger than the working channel which provided enough operation space for the working channel moving to the head, tail, ventral, dorsal, and midline shift, and removed herniated discs. In addition, the gradual use of bone drill and dilating catheter had pushed the soft tissues around guide wire away and reduced the injury rate of peripheral nerve and blood vessel. In this study, 19 patients successfully completed this surgery in group I. Postoperative follow-up showed no neurovascular injury of the patient. Postoperative CT examination showed the diameter of iliac channel matched the expanding drill, and no fractures outside of iliac channel (Fig. 12).

In this study, there was no significant difference in operative time between two groups, which indicated that this surgery did not increase operating time in the circumstance of skilled operation. There was also no significant difference in intraoperative fluoroscopy times between the two groups, but there was an increasing trend in group I (Fig. 10). Hence, we speculated the application of the bone puncture might increase the intraoperative fluoroscopy times, but because of the insufficient samples in this study, there might be some deviation. In early effect of this surgery, there was significant difference in VAS score between before and after surgery, which indicated this surgery could dramatically reduce lower extremity pain of patients. In MacNab score, there were 94.7% of excellent rate in group I and 90% in group R. Yeung and Tsou [17] summarized the excellent rate was 89.3% in the 307 cases with lateral transforaminal endoscopic lumbar discectomy. Schubert [18] treated 588 cases using TESSYS, and the excellent rate was 95.3% in the 2-year follow-up period. This implied this surgery could obtain more good effect when it was used to treat high iliac crest and L5–S1 disc herniation.

The advantages of this surgery in the treatment of high iliac crest and L5–S1 disc herniation are (1) to achieve the cephalad tilt of working channel and remove the dissociated organization; (2) in favor of expanding and molding of the foramen intervertebral; (3) the distance of the center line can be controlled, and decompress the nerve roots to the dorsal side effectively.

Nerve hypoesthesia or allergy postoperatively was known as ‘sunburn syndrome’, which was the most common complication in the operation of percutaneous intervertebral endoscopic and excision of nucleus pulposus [19], and the reason was that the nerve root were stimulated or squeezed during operation. The incidence rate was 5 to 15% and most can be cured by conservative treatment. To avoid this injury, we should note: (1) 0.5% lidocaine was used for local anesthesia for zygopophysis and nerve root, which could reduce pain of winching zygopophysis and avoid nerve root being completely anesthetized. (2) The winching process was strictly monitored by X-ray, and the leading edge of reamer could not exceed the connection of the inner edge of the upper and lower pedicle to avoid nerve injury. (3) All surgical procedures were performed in the spinal canal, so it was necessary to operate under direct vision of intervertebral foramen. And the procedure of removing prolapsed nucleus should be done carefully and gently. (4) Communicating with the patient during surgery to reduce nerve injury.

Limitations of this study: The sample size of this study is small, and the observation time is short. A large sample and follow-up for long term are needed. Disadvantages of the technology: (1) it is difficult to break the hard iliac region; (2) it is difficult to reach the target; (3) potential iliac pain; (4) the possibility of vascular injury, mainly the superior gluteal artery. There may be uncontrolled bleeding during operation; (5) the increase in operating and radiation exposure time; (6) it is difficult to locate iliac point and change the direction of the channel.

## Conclusions

To sum up, the short-term effect of bone puncture technique in transiliac approach to intervertebral endoscopic discectomy for the treatment of L5–S1 intervertebral disc herniation is good, and this is a safe, effective and minimally invasive treatment.

## Abbreviations

PTED: percutaneous transforaminal endoscopic discectomy
VAS: visual analog scale
PELD: percutaneous endoscopic lumbar discectomy
