# Erratum to: Application of transiliac approach to intervertebral endoscopic discectomy in L5/S1 intervertebral disc herniation

**DOI:** 10.1186/s40001-017-0263-z

**Published:** 2017-07-05

**Authors:** Jiayue Bai, Wei Zhang, Yapeng Wang, Jilong An, Jian Zhang, Yapeng Sun, Wenyuan Ding, Yong Shen

**Affiliations:** The Third Hospital of Hebei Medical University, 139#Ziqiang Road, Shijiazhuang, Hebei China

## Erratum to: Eur J Med Res (2017) 22:14 DOI 10.1186/s40001-017-0254-0

Following the publication of the original article [1], the corresponding author stated that he would like to make the following changes:

1. In the abstract, in the first sentence of “Methods” section, “20 patients were diagnosed as non-L5/S1 disc herniation and underwent conventional approach (group R)”, the word “non” is deleted, and thus non-L5/S1 was replaced by L5/S1. Hence the final sentence was “20 patients were diagnosed as L5/S1 disc herniation and underwent conventional approach (group R)”.
2. In the abstract, in the last sentence of “Results” section, “The MacNab score of the last follow-up showed excellent rate (95%) and good rate (90%) in groups I and R, respectively”, we feel the language of this sentence is not accurate and that “The MacNab score of the last follow-up showed excellent and good rate of 94.7% (18/19) and 90% (18/20) in group I and group R, respectively” could be better.
3. In “Materials and methods”, in the second sentence under “Subjects” section, “Twenty patients (12 males and 8 females) with non-L5/S1 disc herniation”, the word “non” is deleted, and thus non-L5/S1 was replaced by L5/S1. Hence the final sentence was “Twenty patients (12 males and 8 females) with L5/S1 disc herniation”.
4. In “Materials and methods”, in the second paragraph of "Inclusion criteria" which is under "Diagnostic criteria" section, the first sentence, “Group R: (1) Single segment without non-L5–S1 segment disc herniation in high iliac crest”, the phrase “without non-L5/S1” has been changed into “with L5/S1”. Hence the final sentence was “Group R: (1) Single segment with L5–S1 segment disc herniation in high iliac crest”.
5. In “Discussion”, in the last sentence of the first paragraph, “The success rate of recurrent patient was about 85%, and below 3% in early period”, we feel the language of this sentence is not accurate and that “The success rate of recurrent patient was about 85%, and recurrence rate in early was below 3%” could be better.
